# Factors Related to Pain and Disability Outcomes After an Internet-Delivered or Physiotherapist-Led Exercise Program for Individuals With Chronic Whiplash Symptoms: Secondary Analysis of a Randomized Controlled Study

**DOI:** 10.2196/67991

**Published:** 2025-05-30

**Authors:** Gunnel Peterson, Stefan Ljunggren, Anneli Peolsson

**Affiliations:** 1 Department of Health Medicine and Caring Sciences, Unit of Physiotherapy, Linköping University, Sjukhusvägen 34, Linköpings Universitet, Linköping, 581 83, Sweden, 46 13281000; 2 Centre for Clinical Research Sormland, Uppsala University, Uppsala, Sweden; 3 Department of Health Medicine and Caring Sciences, Occupational and Environmental Medicine Centre, Unit of Clinical Medicine, Linköping University, Linköping, Sweden

**Keywords:** whiplash, exercise therapy, rehabilitation, spine association, outcome, pain, disability, physiotherapy, exercise, exercising, chronic whiplash, exercise program, whiplash-associated disorders, randomized controlled trial, RCT, secondary analysis, neck pain, neck, visual analogue scale, Neck Disability Index, physical activity, multivariate data analysis, internet-delivered, internet

## Abstract

**Background:**

A neck-specific exercise program has shown sustained clinically important changes in pain and disability for approximately 50% of individuals with chronic whiplash-associated disorders (WAD). However, there is limited information about factors related to treatment response.

**Objective:**

The aim of this study is to identify factors related to changes in disability, neck pain, and physical function after a neck-specific exercise program delivered in 2 different ways for individuals with persistent WAD grade II or III, and to investigate whether any factors could predict those with clinically improved versus not improved disability, pain, and physical function.

**Methods:**

This was a planned secondary analysis of a multicenter prospective randomized controlled trial. Participants (n=140) with persistent (between 6 mo and 5 y from injury) WAD grade II or III were randomized into a 12-week, internet-based neck-specific exercise program (NSEIT) with 4 physiotherapy visits or the same exercise program supervised by a physiotherapist (NSE) twice per week for 12 weeks. Multivariate data analyses and orthogonal partial least squares (OPLS) models were used to investigate change in psychological and physiological factors (independent factors) related to change in the dependent factors: neck-related disability measured with the Neck Disability Index (NDI), neck pain intensity measured with a visual analogue scale, and physical function measured with the Patient-Specific Functional Scale (PSFS). Outcomes were measured at baseline and at 3-month and 15-month follow-up. OPLS discriminant analysis was used to investigate differences between the two groups (NSEIT and NSE) by studying the change scores of the dependent and independent factors. OPLS discriminant analysis was also used to investigate whether background variables and baseline measurements of the independent factors could predict clinically significant improvement in the dependent factors NDI, neck pain, and PSFS.

**Results:**

There were no significant differences between the groups. In both NSEIT and NSE, improvements in the following independent factors were related to improvements in NDI, pain, and PSFS at 3-month and 15-month follow-up: anxiety, depression, cognitive failures, pain catastrophizing, self-efficacy, fear avoidance beliefs, cervical range of motion, headache, and symptom satisfaction (*R*^2^=0.31‐0.37; *Q*^2^=0.25‐0.30; cross-validated ANOVA *P*<.001). No significant OPLS models could be built to distinguish clinically improved versus nonimproved patients as assessed by NDI, neck pain, or PSFS.

**Conclusions:**

Improvements in both psychological and physiological factors were related to improvements in disability, neck pain, and physical function after 12 weeks of NSEIT or NSE. The results indicate that these factors are interrelated and can be improved both with NSEIT and NSE. Known risk factors for poor outcomes of neck disability in WAD, such as low self-efficacy, fear avoidance beliefs, depressive symptoms, and catastrophizing, were improved, and we need to examine other factors not included in this study that can identify those who are not improved after NSEIT or NSE.

## Introduction

Whiplash-associated disorders (WAD) are indirect trauma to the neck, commonly related to a car crash [1], and approximately half of those injured develop chronic disability and pain [1,2]. Chronic WAD will have extensive consequences for the individual living with persistent pain, leading to a burden for the individual and high costs for society [3,4]. Depression, low levels of life satisfaction, and posttraumatic stress have been observed 5 years after whiplash injury [5]. Costs increase as many individuals with chronic WAD are consumers of multiple health services and experience reduced work ability [4,6]. Although the evidence is moderate at best, exercise and patient education are recommended treatments in chronic WAD [7].

Approximately 50% of the included participants in a neck-specific exercise program, conducted by 2 of the authors in this study (GP and AP), with chronic WAD grades II (neck pain and musculoskeletal findings during a physical examination) and III (as grade II but with additional neurological findings) had a sustained clinically important change in pain and disability [8,9]. Despite these promising results, about half of the participants were not clinically improved, demonstrating that chronic moderate-to-severe WAD is difficult to treat successfully. Consequently, there is a need to identify factors related to improvement in disability and pain in order to optimize treatment, discriminating them from factors that may contribute to sustained problems.

An earlier study found that depression, low self-efficacy, and higher disability were associated with a larger pain area in women with chronic WAD [10]. In another study, self-efficacy mediated the association between pain intensity and pain-related disability, and kinesiophobia mediated the association between self-efficacy and pain catastrophizing in acute and subacute WAD [11], which can lead to chronic disability. However, that study did not investigate factors related to improvements after rehabilitation. In studies where interventions included acceptance and commitment therapy or cognitive behavioral therapy, psychological factors had a mediating or predictive effect on pain and/or disability outcome for individuals with chronic WAD [12,13]. Another study found that participation in a neck-specific exercise program was the only significant factor associated with neck pain reduction at 12-month follow-up [14], and neck muscle endurance and perceived work ability were mediators for a reduction in pain [15] in chronic WAD. The treatment of chronic nonmalignant pain is influenced by biological, psychological, and sociological factors [16]. Numerous studies have found factors such as anxiety, catastrophizing, and severe pain to be related to poor treatment outcomes after acute whiplash injury [17].

However, to our knowledge, there is very limited information about factors related to treatment response in chronic WAD, or about the optimization of a neck-specific exercises program delivered via the internet. Internet-based exercise interventions with fewer visits to physiotherapists can increase availability, shorten waiting times, and reduce costs. Internet-based treatment may also strengthen the patient’s own resources for greater autonomy and their ability to conduct long-term self-care [18]. Factors associated with improvements in an internet-based neck-specific exercise program (NSEIT) may differ compared to a neck-specific exercise program supervised by a physiotherapist (NSE) and need to be investigated. Moreover, the ability to predict those who will not recover after NSEIT and NSE is highly relevant to improving treatment in chronic WAD.

The aims of the study were (1) to investigate factors related to change in neck pain and disability after NSEIT or the same exercise program supervised by a physiotherapist (NSE) for individuals with chronic WAD grade II or III and (2) to investigate whether other factors could predict those with clinically improved versus not improved pain and disability after 3 months of NSEIT or NSE.

## Methods

### Overview

The recruitment of participants and the exercise interventions were reported earlier [9] and are briefly described below. This was a planned secondary analysis, based on a multicenter, prospective randomized controlled study [9]. Data were collected from April 6, 2017, until September 15, 2020, and the main outcome data have been published [9]. The participants were recruited from 10 county councils in Sweden.

### Participants

Individuals with chronic (between 6 mo and 5 y from injury) WAD grades II–III [19] with neck symptoms within the first week after a whiplash injury in a 4-wheeled motor vehicle crash were eligible for inclusion. Symptoms emanating from the neck were verified through the participant’s medical history and a physical clinical examination. For additional inclusion and exclusion criteria, see Textbox 1.

Textbox 1. **Inclusion and exclusion criteria.**

**Inclusion**

- Average neck pain last week ≥20 mm on a 0-100 mm visual analogue scale
- Neck disability ≥20%
- Age 18‐63 years
- Daily access to a computer, tablet, or smartphone and the internet
- Sufficient time and motivation to follow the treatment program

**Exclusion**

- Signs of head injury
- Serious physical pathology
- Surgery on the cervical column
- Generalized or more dominant pain elsewhere in the body
- Previous fractures or dislocation of the cervical column
- Other illness or injury that may prevent full participation in the study and/or for which exercises are contraindicated
- Previous severe neck problems that resulted in sick leave for more than 1 month in the year before the current whiplash injury
- Lack of knowledge of the Swedish language
- Diagnosed severe mental illness
- Current alcohol and/or drug abuse

Reasons for the exclusion criteria were as follows: (1) the programs could lead to increased illness or potentially negatively influence other important treatments, (2) another illness or disease made it impossible to perform the programs and/or follow them for three months, (3) the exercises were contraindicated, and (4) participants were unable to understand the information and/or the exercises.

### Ethical Considerations

The study was approved by the regional ethical review board (approval numbers 2016/135‐31, 2016/526‐32, and 2017/45‐32). The study was registered in ClinicalTrials.gov (NCT03022812, initial release 12/20/2016) and the study protocol was published prior to data collection [19]. The original written and informed consent allowed for this secondary analysis without requiring additional consent, and no new data were collected for this study. Deidentification was used to prevent the identification of individuals. The key code is securely stored according to the regulations of the ethics committee and the Swedish Authority for Privacy Protection. The results are presented at the group level, ensuring no connection to individual participants. Participants were not compensated for their involvement in the study, except for traveling cost compensation in accordance with national ethical regulations.

### Recruitment

Information about the study was provided via health care personnel and advertising. Interested participants completed a short survey on the Linköping University website. The patient’s medical history was compiled and a physical examination was carried out before inclusion. Once the study criteria had been met, the participants were included after giving written and oral informed consent. The test leaders were blinded to group allocation. The participants were blinded to group allocation when baseline data were collected [19].

### Randomization

Participants (n=140) were randomized into 2 groups (n=70 in each group) using a computerized block randomization list stratified by sex. After randomization, the treating physiotherapist was contacted by the research team and called the patient to the first appointment.

Before the start of the study, all treating physiotherapists received oral and written information and a day of practical training from the project leaders. A detailed description of the NSEIT and NSE exercise program has been published [9].

### NSEIT Group

In the NSEIT group, the participants underwent a 12-week exercise program with 4 visits to the physiotherapist (at weeks 1, 2, 3, and 7) to be introduced to the exercises, to progress the exercises, and for follow-up to ensure correct performance. The patient had access to the internet-based program on a website. The program included photos and videos of the exercises, information about the musculoskeletal anatomy of the neck and about neurophysiological and neurobiological pain, and strategies for dealing with neck pain relapse. The exercises were initially targeted to activate the deep neck muscles and increase postural control, continuing with an individually tailored progression of neck muscle endurance exercises [9,19]. At the end of the treatment period, the participants were encouraged to continue doing the exercises 2-3 times per week and to include neck-specific exercises (for additional details, see [9]).

### NSE Group

In the NSE group, participants received the same information and exercises as the NSEIT group, but in visits to the physiotherapy clinic twice per week (a maximum of 24 sessions) [9,19].

### Patient and Public Involvement

Patients were involved in developing the questionnaires before recruitment to the study started. The questionnaires were tested by 3 patients to ensure the inclusion of relevant questions and readability. Patient satisfaction with the exercises, information, tests, and intervention (NSEIT or NSE) were measured postintervention.

Adverse events could be reported to the project leaders directly by participants, the test leaders, or the treating physiotherapists (for details, see [9]).

### Predictor Variables and Outcome Measures Used in Multivariate Modeling

Measurements were collected via questionnaires at baseline and at 3-month and 15-month follow-ups. The following variables were used in the multivariate modeling.

#### Background Variables

The following variables were only collected at baseline: age (years), sex (male/female), smoking (yes/no), level of education, pain duration (months since injury), WAD grade II and III according to the Quebec Task Force classification [20], and expectations of the intervention on a 0 to 10 scale (with higher scores indicating stronger belief that the treatment would help) [21].

#### Dependent Factors

The Neck Disability Index (NDI*)* measures self-perceived disability in everyday life because of neck pain, including 10 items of neck-related disability, which are summed and transformed into a percentage (0%=no disability; 100%=highest score for disability) [22].

Current neck pain was measured on a visual analogue scale (VAS) from 0‐100 mm, where 0=no pain and 100=worst imaginable pain intensity [23].

The Patient-Specific Functional Scale (PSFS*)* measured level of physical function, graded from 0 (unable to do the activity) to 10 (functional level equal to preinjury status), regarding self-perceived physical activity, leisure time, and work [24].

#### Independent Factors

Measures of factors considered as potentially related to change in pain and disability were selected based on a review of the literature and the clinical experience of 2 specialist physiotherapists with over 15 years of experience managing patients following a whiplash injury:

- Self-reported pain medication use (“Do you take pain medication?” Yes/no).
- Symptom satisfaction was measured on a 7-point scale from 1=happy about symptoms to 7=unhappy about symptoms [25].
- Exercise level rating of daily activities and sports (from 1=inactivity to 4=high activity level) [26].
- Dizziness at rest and while in motion and unsteadiness and headache intensity were measured on VAS scales from 0 mm=no dizziness/unsteadiness or headache to 100 mm=extreme dizziness/unsteadiness or headache [23,27].
- The Post-Traumatic Stress Disorder Checklist (the specific trauma version, PCL-S) measures posttraumatic stress reactions and consists of 4 subscales (re-experience, avoidance, negative alterations in cognition and mood, and hyperarousal), with a total score ranging from 0=no stress reaction to 80=extreme stress [28].
- Fear Avoidance Beliefs Questionnaire measures the individual’s fear of pain and consequent avoidance of physical activity because of their fear. It consists of 2 subscales (Physical Activity subscale [4 items] and Work subscale [7 items]), with a total score ranging from 0 to 66, where higher scores indicate higher fear avoidance beliefs [29].
- The Hospital Anxiety and Depression Scale gives an anxiety score (from 0=no anxiety to 21=maximal anxiety) and a depression score (from 0=absent depression to 21=maximal depression) [30].
- The Pain Catastrophizing Scale measures the extent of catastrophizing according to the 3 subscales (rumination, magnification, and helplessness), with a total score range of 0‐52, with higher scores indicating higher pain catastrophizing [31].
- The Self-Efficacy Scale measures self-perceived performance of 20 everyday functional tasks in spite of pain, with total scores ranging from 0 to 200, where higher scores indicate higher perceived self-efficacy [32].
- The Cognitive Failures Questionnaire measures behavioral problems associated with attentiveness and memory in everyday life, with the total score ranging from 0 to 100, where a higher score indicates a greater frequency of cognitive failures [33].
- The EuroQol quality-of-life instrument version with 5 dimensions and 3 response levels (EQ-5D-3L) and EuroQol health-related quality of life measure using a VAS (EQ-VAS) measure health-related quality of life. EQ-5D-3L scores range from −0.624 to 1 and EQ-VAS scores range from 0 to 100 mm, with higher values indicating better health-related quality of life [34].

The following physiological measures were collected by the test leader at baseline and 3-month and 15-month follow-up:

- Dynamic hand strength was measured in kilograms using the Jamar dynamometer [35].
- Ventral and dorsal neck muscle endurance (NME*)* was registered in seconds [36].
- Active range of cervical motion in sagittal, lateral, and frontal planes was recorded in degrees using the cervical range-of-motion device [37].
- The Cranio-Cervical Flexion test was used to measure the action of the deep cervical flexor muscles, registered in mm Hg [38].
- Balance test: standing on one leg with eyes closed was measured in seconds [27].

### Statistical and Multivariate Data Analysis

Multivariate data analysis was used to investigate the relationships between the variables using SIMCA software (v.18.0.0.372; Sartorius Stedim Data Analytics AB). For this, orthogonal partial least squares (OPLS) and OPLS discriminant analysis (OPLS-DA) were used. In general terms, these methods are linear regression methods that model the association between predictors (X) and response (Y) variables. They do this by summarizing the information in the original variables into a few virtual variables (components) that are selected to maximize the covariance between the X and Y variables. This allows the identification of the association between many X and Y variables simultaneously. The methods also separate variation in predictors that are either predictive for the response variables or not (orthogonal). This minimizes the issue of collinearity seen with classical regression models.

First, the differences between patients randomized to either NSEIT or the same exercises and information at a physiotherapy clinic (NSE) [9] were investigated by studying the changes (change scores) of variables between the 3 time points (baseline to 3 mo, baseline to 15 mo, and 3 mo to 15 mo) using OPLS-DA. Both the dependent factors disability (NDI), neck pain (VAS), and physical function (PSFS) and the independent factors (defined above) were included as X variables to investigate which of the variables may explain differences between NSE and NSEIT.

Second, OPLS models were used to investigate the relationship between changes for the dependent and independent factors at the 3 time points (baseline to 3 mo, baseline to 15 mo, and 3 mo to 15 mo). Y variables were set to the change scores for the dependent factors NDI, neck pain, and PSFS (using the average of the 3 PSFS components physical activity, leisure time, and work). X variables included all independent factors and all background variables detailed previously. A positive change score indicates improvement (eg, a higher change score in neck pain indicates less pain) and a negative change score indicates deterioration. To identify which of the X variables (independent factors) were most important for the changes in the Y variables (dependent factors; NDI, neck pain, and PSFS), those independent factors in a first model that had a variable influence on projection value of at least 1.0 and a variable influence on projection value greater than the standard error were included in a second OPLS model with one predictive and one orthogonal component.

Third, multivariate models using OPLS-DA were used to investigate whether background variables and baseline measurements of any of the independent factors could predict clinically significant improvement in the dependent factors NDI, neck pain, and PSFS. Individuals with clinically improved NDI, neck pain, and PSFS (value change NDI ≥7, neck pain ≥50% improvement, PSFS ≥2-point improvement) [22,23,39] were compared to individuals with no improvement or deteriorated values on these outcome measures.

All multivariate models were assessed using the fit of the data to the model (*R*^2^) and the predictability of the model fit on new data (*Q*^2^); the significance of group separation was assessed using cross-validated analysis of variance (CV-ANOVA) [40]. The maximum value for each (*R*^2^ and *Q*^2^) is 1.0 and symbolizes a perfect model. The loadings (weights representing the variables) were used for interpretation of the association between the X (independent factors) and Y (dependent factors) variables.

Sample size and power calculations were calculated based on the primary outcome measure (NDI) in the previous randomized controlled study [8]. To detect a between-group noninferiority margin of 7% with 1-sided *α*=.025 and *β*=.8, a total of 47 participants were needed in each group. To account for dropouts, 70 participants were included in each of the 2 groups (140 in total) [9]. Compliance with the exercise program (NSEIT, NSE) was defined as at least 50% self-reported completion of the exercises.

## Results

### Overview

**Figure 1.**
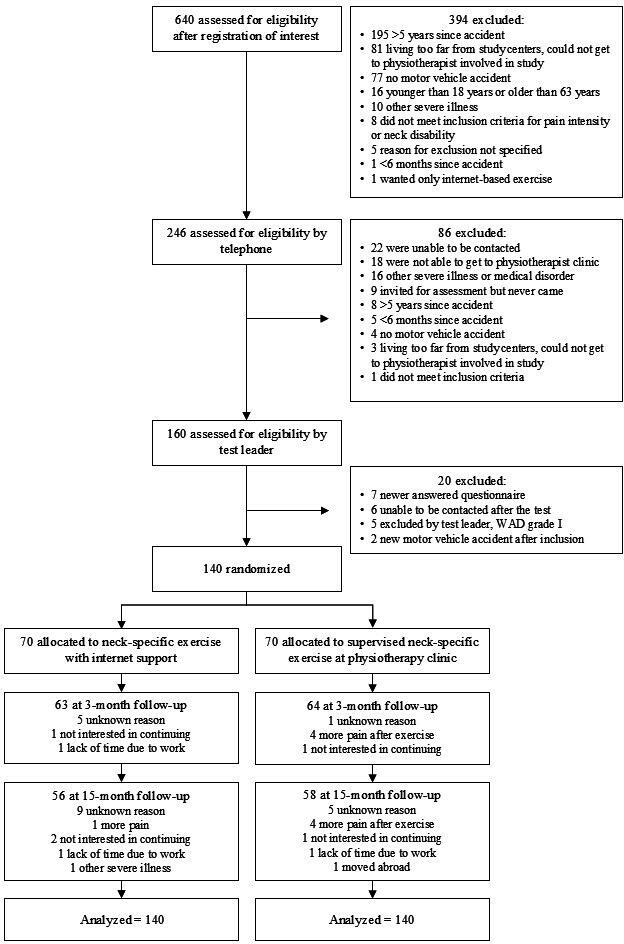
Flowchart of participants in this study. WAD: whiplash-associated disorder.

Baseline characteristics of the participants are shown in Table 1. In the NSEIT group, of the initial 70 participants, 63 (90%) and 56 (80%) participants completed the 3-month and 15-month questionnaires, respectively. In the NSE group, of the initial 70 participants, 64 (91%) and 58 (83%) completed the 3-month and 15-month questionnaires, respectively (Figure 1).

**Table 1.** Baseline characteristics of participants with whiplash-associated disorders grade II and III, by treatment group.

	NSEIT^a^ (n=70)	NSE^b^ (n=70)
Age (years), mean (SD)	40.4 (11.6)	40.5 (11.4)
Sex, female, n (%)	55 (79)	55 (79)
Months since injury, mean (SD)	27.4 (21.0)	25.2 (15.5)
**Whiplash-associated disorders, n (%)**		
Grade II^c^	46 (66)	43 (61)
Grade III^d^	24 (34)	27 (39)
**Educational level, n (%)**		
Elementary school	1 (1)	0 (0)
Upper secondary school	30 (43)	39 (57)
University	35 (50)	27 (39)
Other	4 (6)	3 (4)
Marital status, married or cohabiting, n (%)	52 (74)	53 (76)
Previous treatment, yes, n (%)	62 (89)	66 (94)
**Use of analgesic drugs, n (%)**		
Yes	57 (81)	60 (86)
No	12 (17)	10 (14)
**Sick leave, n (%)**		
No	61 (87)	62 (89)
Sick leave, part-time	6 (9)	5 (7)
Sick leave, full-time	3 (4)	3 (4)
Disability (Neck Disability Index, 0%‐100%)	39.4 (12.2)	36.6 (10.8)
**Neck pain intensity, mean (SD)**		
Neck pain now, VAS^e^ (0‐100 mm)	34.6 (21.4)	39.7 (22.4)
Neck pain average previous week, VAS (0‐100 mm)	44.9 (20.6)	47.6 (21.7)
**Patient-Specific Functional Scale, mean (SD)**		
Work (0‐10)	3.9 (2.1)	3.6 (2.3)
Leisure time (0‐10)	3.7 (2.1)	3.6 (2.3)
Activity (0‐10)	3.2 (2.5)	3.5 (2.8)

a NSEIT: internet-based neck-specific exercise program.

b NSE: neck-specific exercise program supervised by a physiotherapist.

c Whiplash-associated disorders grade II: neck pain and musculoskeletal findings during a physical examination.

d Grade III: like grade II but with additional neurological findings.

e VAS: visual analogue scale.

During the 12-week intervention period, the participants’ self-reported compliance with the exercise program was higher in the NSE group (59/64, 92%) than in the NSEIT group (46/63, 73%; *P*=.006). No adverse events were reported.

### Between-Group Differences

No significant OPLS-DA models could be built to separate patients receiving NSEIT from those receiving NSE (baseline to 3 mo: *R*^2^=0.22, *Q*^2^=–0.12; baseline to 15 mo: *R*^2^=0.22, *Q*^2^=0.01; 3 mo to 15 mo: *R*^2^=0.17, *Q*^2^=–0.10; all models CV-ANOVA *P*>.99). There were also no differences between the NSEIT and NSE groups regarding independent factors associated with improvements in the dependent factors NDI, neck pain, and PSFS (Figures S1-S3 in Multimedia Appendix 1).

### Factors Related to Improvements in Disability, Neck Pain, and Physical Function

No background factors were found to be associated with improvements in NDI, neck pain, or PSFS in any of the multivariate models.

#### Improvement From Baseline to 3-Month Follow-Up

**Figure 2.**
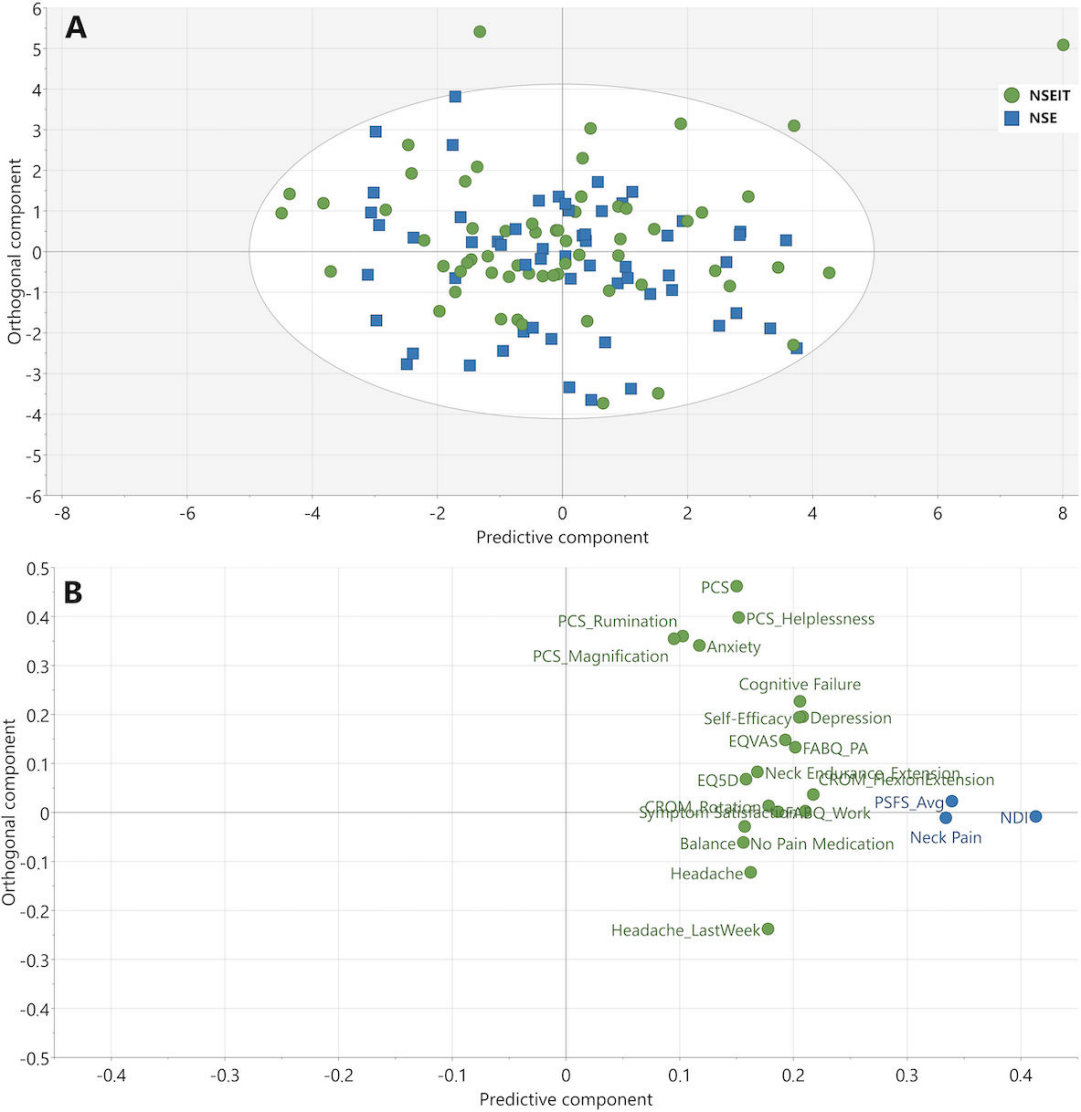
OPLS model depicting changes in independent factors from baseline to 3 months. (A) Score plot of the participating patients. The color corresponds to grouping in either NSE (blue squares) or NSEIT (green circles). There were no differences between the NSEIT and NSE groups. (B) Loading plot of analyzed variables, showing the contribution of each independent factor to the OPLS component underlying the participant separation in the score plot (A). The x-axis depicts the most important predictive component for separating participants based on improvements in NDI, neck pain, and PSFS (blue circles) in both the NSEIT and NSE groups (*R*^2^=0.31, *Q*^2^=0.25, cross-validated ANOVA *P*<.001). CROM: cervical range of motion; EQ5D: EuroQol health-related quality-of-life scale; EQ-VAS: EuroQol visual analogue scale; FABQ: Fear Avoidance Beliefs Questionnaire; NDI: Neck Disability Index; NSE: neck-specific exercise program supervised by a physiotherapist; NSEIT: internet-based neck-specific exercise program; OPLS: orthogonal partial least squares; PCS: Pain Catastrophizing Scale; PSFS_Avg: Patient-Specific Functional Scale average (the average of the 3 PSFS components physical activity, leisure time, and work).

At 3 months compared to baseline, 20 independent factors were identified as associated with improvements in NDI, neck pain, or PSFS (*R*^2^=0.31, *Q*^2^=0.25, CV-ANOVA *P*<.001; Figure 2).

#### Improvement From Baseline to 15-Month Follow-Up

**Figure 3.**
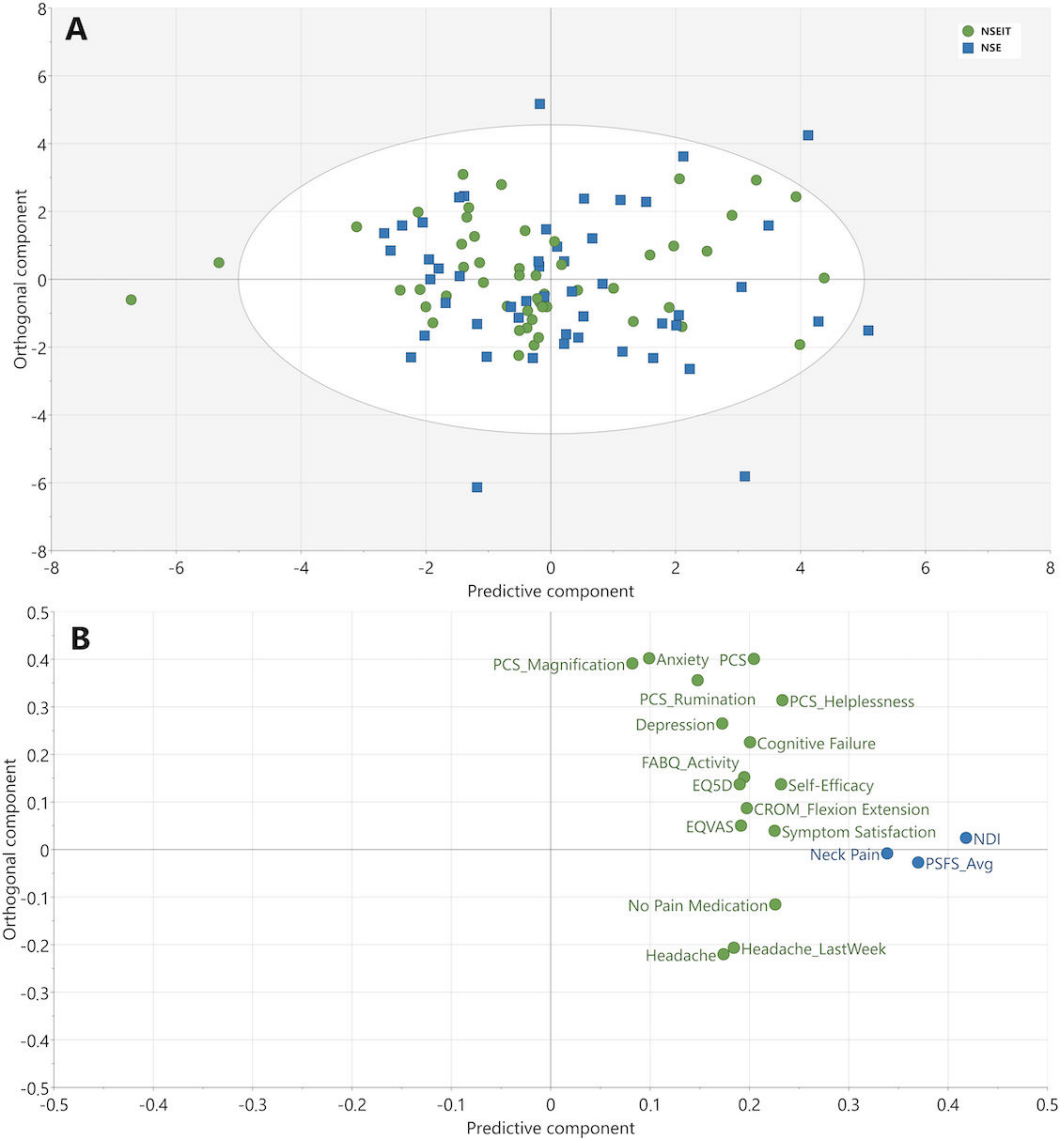
OPLS model depicting changes in independent factors from baseline to 15 months. (A) Score plot of the participating patients. The color corresponds to grouping in either NSE (blue squares) or NSEIT (green circles). There were no differences between the NSEIT and NSE groups. (B) Loading plot of analyzed variables, showing the contribution of each independent factor to the OPLS component underlying the participant separation in the score plot (A). The x-axis depicts the most important predictive component for separating participants based on improvements in the primary outcomes NDI, neck pain, and PSFS (blue circles; *R*^2^=0.37, *Q*^2^=0.30, cross-validated ANOVA *P*<.001 for NDI and neck pain and *P*<.01 for PSFS). CROM: cervical range of motion; EQ5D: EuroQol health-related quality-of-life scale; EQ-VAS: EuroQol visual analogue scale; FABQ: Fear Avoidance Beliefs Questionnaire; NDI: Neck Disability Index; NSE: neck-specific exercise program supervised by a physiotherapist; NSEIT: internet-based neck-specific exercise program; OPLS: orthogonal partial least squares; PCS: Pain Catastrophizing Scale; PSFS_Avg: Patient-Specific Functional Scale average (the average of the 3 PSFS components physical activity, leisure time, and work).

At 15 months compared to baseline, 16 independent factors were identified as associated with improvements in NDI, neck pain, and PSFS (*R*^2^=0.37, *Q*^2^=0.30, CV-ANOVA *P*<.001 for NDI and neck pain and *P*<.01 for PSFS; Figure 3).

#### Improvement From 3-Month to 15-Month Follow-Up

**Figure 4.**
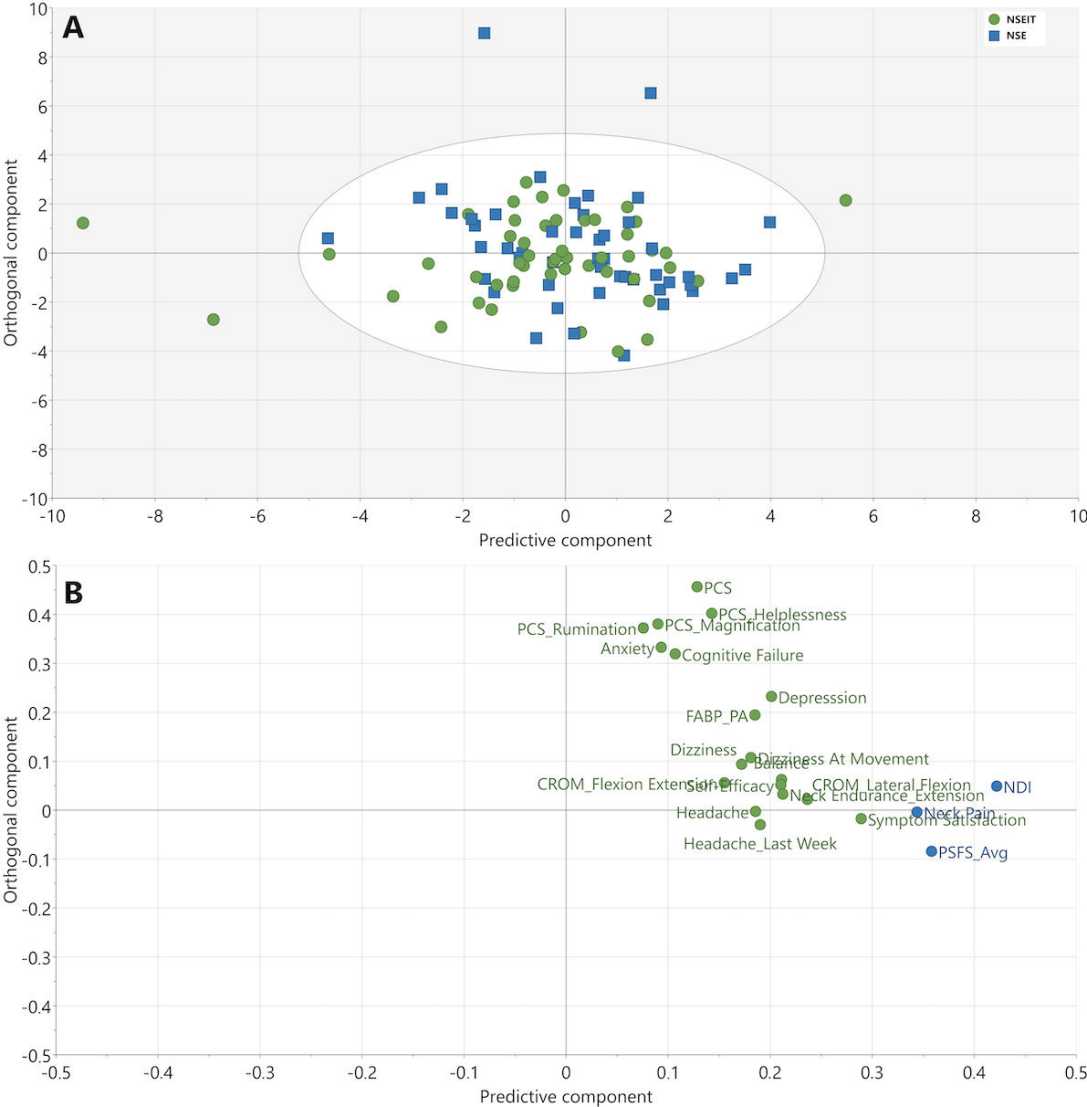
OPLS model depicting changes in independent factors from 3 months to 15 months. (A) Score plot of participating patients. The color corresponds to grouping in either NSE (blue squares) or NSEIT (green circles). There were no differences between the NSEIT and NSE groups. (B) Loading plot of analyzed variables, showing the contribution of each independent factor to the OPLS component underlying the participant separation in the score plot (A). The x-axis depicts the most important predictive component for separating participants based on improvements in the primary outcomes NDI, neck pain, and PSFS (blue circles; *R*^2^=0.34, *Q*^2^=0.28, cross-validated ANOVA *P*<.001). CROM: cervical range of motion; EQ5D: EuroQol health-related quality-of-life scale; EQ-VAS: EuroQol visual analogue scale; FABQ: Fear Avoidance Beliefs Questionnaire; NDI: Neck Disability Index; NSE: neck-specific exercise program supervised by a physiotherapist; NSEIT: internet-based neck-specific exercise program; OPLS: orthogonal partial least squares; PCS: Pain Catastrophizing Scale; PSFS_Avg: Patient-Specific Functional Scale average (the average of the 3 PSFS components physical activity, leisure time, and work).

At 15 months compared to 3 months, 18 independent factors were identified to be associated with improvements in NDI, neck pain, and PSFS (*R*^2^=0.34, *Q*^2^=0.28, CV-ANOVA *P*<.001; Figure 4).

#### Variable Loadings for the 3 Models

**Figure 5.**
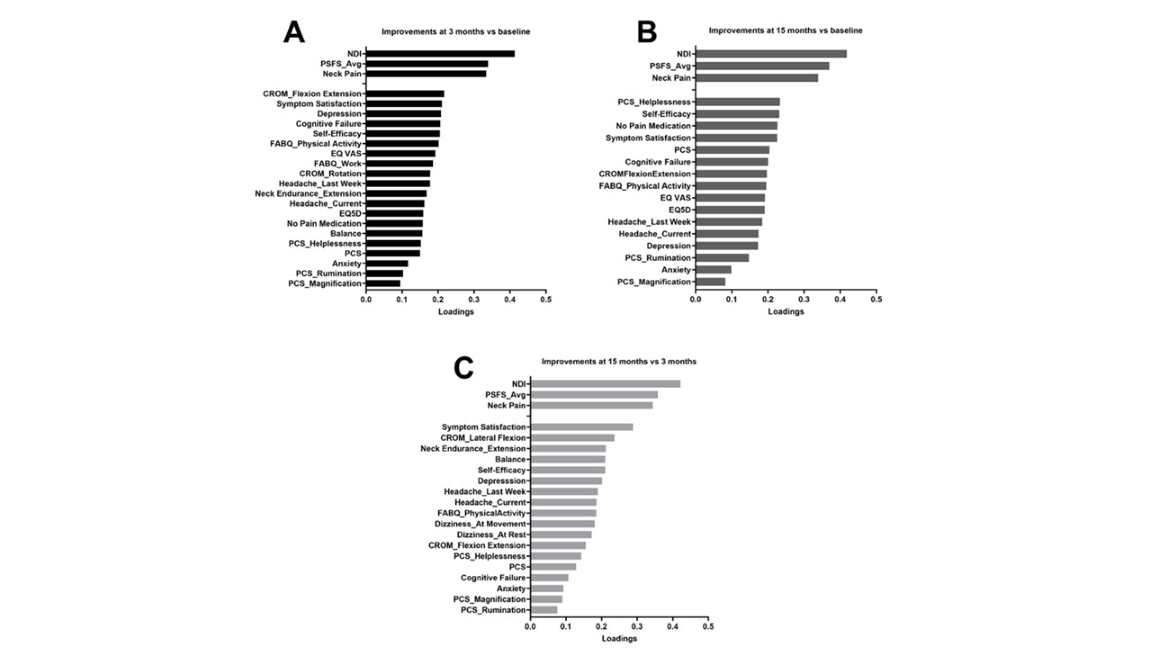
Loadings (contribution of each independent factor) obtained through OPLS after selecting outcome measures with variable influence on projection greater than 1.0. The three models (A-C) show the association between independent factors for improvements in NDI, neck pain, and PSFS at different time points. (A) Improvements between baseline and 3-month follow-up. (B) Improvements between baseline and 15-month follow-up. (C) Improvements between 3-month and 15-month follow-up. A higher value for the loadings indicates it is more important in the models. CROM: cervical range of motion; EQ5D: EuroQol health-related quality-of-life scale; EQ-VAS: EuroQol visual analogue scale; FABQ: Fear Avoidance Beliefs Questionnaire; NDI: Neck Disability Index; NSE: neck-specific exercise program supervised by a physiotherapist; NSEIT: internet-based neck-specific exercise program; OPLS: orthogonal partial least squares; PCS: Pain Catastrophizing Scale; PSFS_Avg: Patient-Specific Functional Scale average (the average of the 3 PSFS components physical activity, leisure time, and work).

The contribution of each variable (loadings) to the predictive component (x-axis) for the 3 models (Figures 1-3) is visualized in Figure 5. Thirteen of the 23 identified independent factors were common for all 3 models and included both psychological and physiological factors. The most important factors, as determined by a higher loading, that were related to improvements in NDI, neck pain, and PSFS were as follows: anxiety and depression, cognitive failure, pain catastrophizing, self-efficacy, fear avoidance beliefs, cervical range of motion, headache last week, current headache, and symptom satisfaction. Factors only found in 1 or 2 models were fear avoidance beliefs subscale work, balance (standing on one leg), cervical motion (lateral flexion and rotation), neck muscle endurance (extension), taking no pain medication, dizziness at rest and at movement, and health-related quality of life (Figure 5).

The 5 most important independent factors related to changes in NDI, neck pain, and PSFS between baseline and 3-month follow-up were improvement in cervical range of motion (flexion/extension), symptom satisfaction, depression, cognitive failure, and self-efficacy (Figure 5A).

The 5 most important independent factors related to changes in NDI, neck pain, and PSFS between baseline and 15-month follow-up were improvement in pain catastrophizing (helplessness), self-efficacy, taking no pain medication, symptom satisfaction, and pain catastrophizing (total score; Figure 5B).

The 5 most important independent factors related to changes in NDI, neck pain, and PSFS between 3-month and 15-month follow-up were improvement in symptom satisfaction, cervical range of motion (lateral flexion), NME (extension), balance, and self-efficacy (Figure 5C).

### Baseline Factors Determining Clinically Improved Versus Nonimproved Patients in Disability, Neck Pain, and Physical Function

Using OPLS-DA modeling, no significant models could be built to separate those patients whose NDI, neck pain, or PSFS was clinically improved from those with no improvement or showing deterioration based on background variables and baseline measurements of any of the independent factors (Figures S4-S9 in Multimedia Appendix 1).

## Discussion

### Principal Findings

This secondary analysis of a prospective randomized study investigated factors related to change in neck pain and disability (including functional ability) after a NSEIT or NSE exercise program for individuals with chronic WAD grade II or III.

The multivariate OPLS-DA models showed no significant differences between NSEIT and NSE. Thus, factors related to changes in pain and disability were the same in both groups, independent of how the exercise program was delivered. On the other hand, the multivariate OPLS models of improvements in neck pain and disability between the 3 time points (baseline to 3 mo, baseline to 15 mo, and 3 mo to 15 mo) indicated a number of related factors in both NSEIT and NSE. In total, 23 factors were identified and 13 of them featured in all 3 time points. However, neither the baseline factors nor changes in the independent factors could predict which patients were improved versus nonimproved in pain and disability.

Improvements in psychological and physiological factors were associated with an improvement in disability, pain, and physical function in both groups (NSEIT and NSE). It seems reasonable to assume that the effect may be related to the neck-specific exercise intervention itself, regardless of whether it was internet-based or physiotherapist-supervised, because the neck-specific exercise and information program were identical in NSEIT and NSE. The 4 face-to-face visits in NSEIT included examination, an introduction to performing the exercises, progression of exercises, and follow-up. A possible explanation is that 4 visits to the physiotherapist combined with the internet-based program enabled these patients to feel confident and skilled enough to perform the exercises to the same extent as participants in the NSE group, who had many more visits to the physiotherapist.

In our study, participants in both groups described having increased knowledge regarding WAD, a stronger neck, and fewer symptoms [41]. Moreover, no significant differences were seen between NSEIT and NSE for primary or secondary outcomes [9,42].

The interpretation of the multivariate OPLS models was based on the weights (loadings) reflecting the relationship between dependent factors (NDI, neck pain, PSFS) and independent factors. Thus, independent factors with higher loadings were more closely related to improvements in NDI, neck pain, and PSFS compared to outcomes with lower loadings in the same model (Figure 5). Improvements in self-efficacy and symptom satisfaction were prominent in all 3 models (baseline to 3 mo, baseline to 15 mo, and 3 mo to 15 mo). Neck-specific exercises have been shown to improve neck-related function [42] and, in combination with the patient education in our NSEIT/NSE program, the exercises may lead to an enhanced ability to perform everyday functional activities, as the improvements in NDI and PSFS indicate [9].

### Comparison to Prior Work

Psychological factors such as low self-efficacy, fear avoidance beliefs, depressive symptoms, and catastrophizing have earlier been shown to be predictors of a poor outcome of neck disability in chronic WAD [43-47], but they are also shown to be improved after 3 months of neck-specific exercise [48]. One of the most important factors to improve self-efficacy is personal mastery experiences, that is, the ability to successfully perform a task [49]. When individuals in the NSEIT and NSE groups could perform activities with less pain, their self-efficacy and symptom satisfaction increased. A painful stimulus, such as neck pain, can trigger catastrophic thinking, fear-avoidance beliefs, and avoidance behaviors, resulting in more pain and depression and leading to chronic disability as described by Vlaeyen and Linton [50] in their fear-avoidance model. Our neck-specific exercise program included information on whiplash injury, outlining why pain can persist even after potential injuries to muscles or ligaments have healed, and highlighting the importance of exercising the neck muscles. The exercises should not exacerbate neck pain but should be progressed over time, leading to the activity level recommended by the World Health Organization, as described in detail previously [9].

Physical activities and exercises are highly effective for improving symptoms of depression [51]. This may explain the association between improvements in NDI and PSFS (physical function) and depressive symptoms in this study. A speculative explanation is that the effect of our neck-specific exercise program is related to mutually positive associations. Physical function and self-efficacy increased—while pain, fear avoidance, and depression decreased—as a result of the exercise program. Anxiety and pain catastrophizing were included in the 3 models but had a lower influence on the dependent factors (neck pain and disability). Improvement in cognitive failures was also related to an improvement in pain and disability in all 3 models. Previous studies have shown that cognitive failures and attention problems are common and disabling when living with pain [52], a finding that is not limited to subjective reports, as these patients also fail in standardized cognitive tests [53]. However, another study found the relationship between chronic pain and cognitive tests in whiplash inconclusive [54]. Coexisting brain injury due to the whiplash trauma may be another reason for cognitive failures independent of pain [53], although individuals with brain injury were excluded in this study. Our results indicate that improvement in pain and disability after NSEIT or NSE was to some extent related to improvement in anxiety, pain catastrophizing, and cognitive failures. A causal relationship cannot be established in this study, but the findings may be of importance for improved rehabilitation in chronic WAD.

Background variables and baseline values of the independent factors in our study could not separate individuals with clinical improvement versus nonimprovement in disability, neck pain, and physical function. Moreover, no background variables were found to be associated with improvements in NDI, neck pain, or PSFS. A possible reason is that we failed to include the most relevant factors in the analyses despite the inclusion of many variables in the OPLS and OPLS-DA models. However, we included background variables and independent factors that were previously reported in the literature to be important in predicting and relating to disability and pain in WAD and chronic pain [10,11,14,43,44,46,47,55-57]. Nonetheless, there are conflicting results in earlier studies regarding the importance of some of the background variables and baseline values of the independent factors to pain intensity in chronic pain [55]. In chronic WAD, female gender, older age, and WAD grade III were related to nonrecovery in one study [56], while another study found that pain-related catastrophizing explained the variance in disability in patients with chronic WAD [44]. In contrast, we have previously found that no background variables were predictive of pain reduction in chronic WAD at 1-year follow-up [14]. However, higher baseline NDI score, absence of dizziness, and higher patient expectations, but not psychological factors, were associated with disability reduction [14]. We have also previously found that neck muscle endurance and perceived work ability were mediators for reduction in pain [15] in chronic WAD, and we identified a lack of improvement in ventral NME as a factor for ongoing dizziness in WAD after an exercise intervention [57]. The participants in this study had higher neck muscle endurance at baseline [42] compared to earlier results [58] and they improved more at the 3-month follow-up [42,58]. Higher neck muscle endurance may reduce disability and pain, which in turn can influence other factors and may explain the discrepancy between our results and other studies [12,44,56].

We recently reported that between 47% and 74% of participants with chronic WAD grade II and III demonstrated sustained improvement in disability, pain, and physical function after 3 months of NSEIT or NSE [9], but we failed to identify factors associated with responders and nonresponders. Differences in analysis methods may explain the discrepancy between the present results and other studies [12,44,56]. An interesting result in this study was that neither psychological factors such as low self-efficacy, depression, and fear avoidance, nor background characteristics such as older age, low disability level, or WAD grade III, could predict nonresponders. Further studies should include factors related to possible tissue damage or altered function in chronic WAD, but one problem is that there is still a lack of reliable and effective diagnostic tools.

The results indicate that we need to go further to be able to improve our understanding of chronic WAD and to optimize treatment. Biomedical and/or psychosocial factors not investigated in this study are likely to be important for identifying nonresponders to NSEIT and NSE in chronic WAD.

### Strengths and Limitations

This is the first study investigating factors related to pain and disability after an internet-based exercise program for chronic WAD grade II and III patients and, to our knowledge, factors related to the outcome of PSFS have not previously been reported for chronic WAD.

The strengths of multivariate OPLS analysis are the ability to include many variables and observations and the fact that several response variables (dependent factors) can be modeled together. OPLS can also cope with multicollinearity and missing values, which is a major advantage compared with traditional regression models. OPLS models use latent variables to model the data; these latent variables, which are linear combinations of the original variables, reduce the dimensions and minimize the issue of multicollinearity. Furthermore, OPLS separates the predictive variation (related to the response variable) from the orthogonal variation (unrelated to the response variable), thus focusing on the most relevant information, which simplifies the model and improves interpretability [59-61]. Moreover, the benefits of using multivariate statistics in this analysis are that variables can be scaled and centered on the mean, implying that each variable will have the same potential to affect the model, and that a patient or cluster of patients can be related to a variable profile rather than a single variable.

There are several limitations in this study. First, in the OPLS analyses, all 3 models (baseline to 3 mo, baseline to 15 mo, and 3 mo to 15 mo) showed relatively low explained variance, with the highest explanation level for the baseline to 15-month follow-up (*R*^2^=0.37, *Q*^2^=0.30). The goodness of fit of a model varies depending on the type of data and the specific application. For example, spectroscopic calibration models require values close to 1.0. However, while high *R*² and *Q*² values are desirable, they must be interpreted in the context of the specific application and data type [62]. Lower values indicate a less robust fit and predictive ability but might be acceptable, for example, in OPLS models that include self-reported psychological symptoms. Second, the results of this study are only generalizable to those individuals who fulfill the study criteria and are motivated to participate in exercise, but not to all individuals with WAD. Moreover, long-term effects can be influenced by various factors. For example, consistently measuring outcomes over time can introduce bias and affect the reliability of the results due to changes in the patient’s life situation. Additionally, dropouts commonly increase over time. Third, only individuals who understood the Swedish language were included, and the age range was limited to 18‐63 years. Thus, this study excluded minorities from a different background who did not understand Swedish and the results cannot be generalized to those aged under 18 or over 63 years.

### Conclusions

After 3 months of NSEIT or NSE, improvements in neck pain and disability were related to improvements in 23 other physical and psychological factors. However, the findings should be interpreted with caution, as the results showed relatively low explained variance. No recommendations can be given for which individuals will benefit from NSEIT or NSE, due to the study’s inability to distinguish between clinically improved and nonimproved participants regarding neck pain and disability. Further research is warranted to improve our understanding of those who are improved or not improved after 3 months of NSE or NSEIT.

## Supplementary material

10.2196/67991 Multimedia Appendix 1 Orthogonal partial least squares models.

## Abbreviations

CV-ANOVA: cross-validated analysis of variance
EQ-5D-3L: EuroQol quality-of-life instrument version with 5 dimensions and 3 response levels
EQ-VAS: EuroQol health-related quality of life measure using a visual analogue scale
NDI: Neck Disability Index
NME: neck muscle endurance
NSE: neck-specific exercise program supervised by a physiotherapist
NSEIT: internet-based neck-specific exercise program
OPLS: orthogonal partial least squares
OPLS-DA: orthogonal partial least squares discriminant analysis
PSFS: Patient-Specific Functional Scale
VAS: visual analogue scale
WAD: whiplash-associated disorders
