# Supplementary material for: Factors Related to Pain and Disability Outcomes After an Internet-Delivered or Physiotherapist-Led Exercise Program for Individuals With Chronic Whiplash Symptoms: Secondary Analysis of a Randomized Controlled Study
Source: JMIR Hum Factors. 2025 May 30;12:e67991. doi: 10.2196/67991 (PMC12143857; doi:10.2196/67991)
Supplement: Multimedia Appendix 1 [file humanfactors-v12-e67991-s001.pdf]

Supplemental Material

Supplementary Figure 1. Comparison between the NSEIT and NSE group baseline to 3 months follow-up

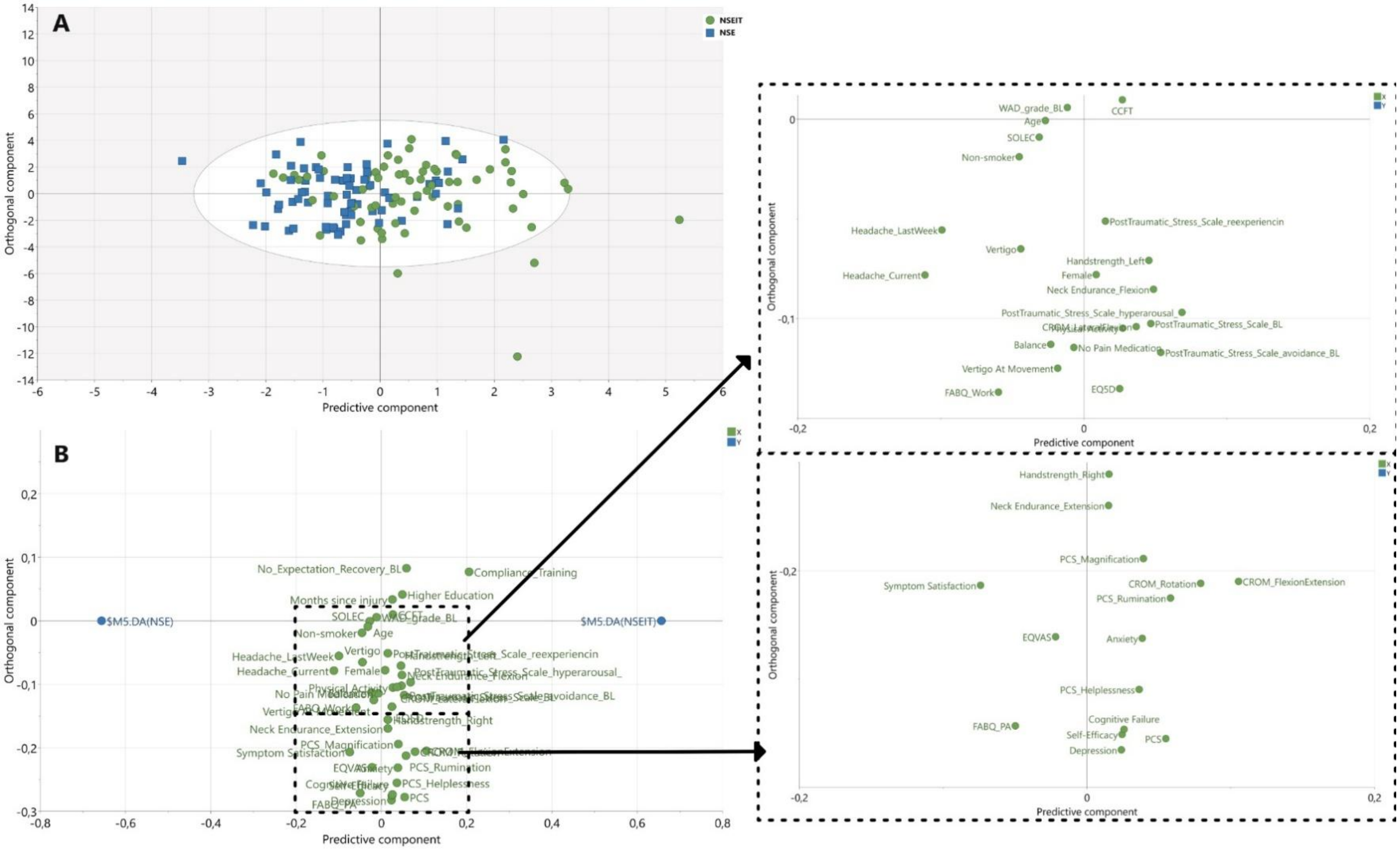

Orthogonal partial least squares discriminant analysis (OPLS-DA) model of NSE and NSEIT including the three dependent variables (NDI, neck pain and PSFSAVG) and the change score (baseline to 3 months) of all independent variables. No clear separation of the NSEIT and NSE groups were seen. The x axis depicts the predictive component most important for separating groups, while the y axis represents the orthogonal component showing in-group variation, R2 = 0.22, Q2 = -0.12, CV-ANOVA p-value = 1.

Supplementary Figure 2. Comparison between the NSEIT and NSE group baseline to 15 months follow-up

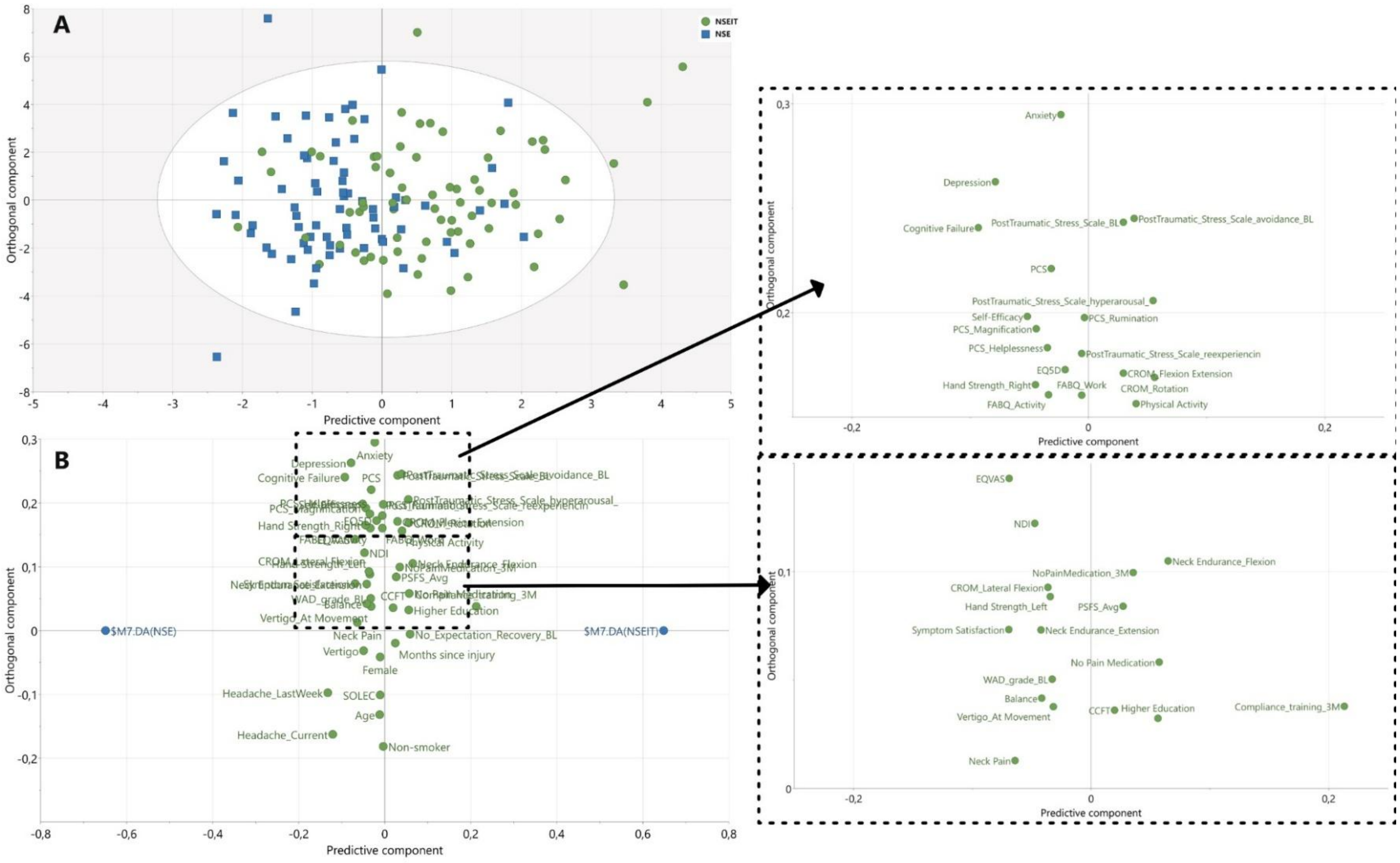

Orthogonal partial least squares discriminant analysis (OPLS-DA) model of NSE and NSEIT including the three dependent variables (NDI, neck pain and PSFSAVG) and the change score (baseline to 15 months) of all independent variables. No clear separation of the NSEIT and NSE groups could be seen. The x axis depicts the predictive component most important for separating groups, while the y axis represents the orthogonal component showing in-group variation, R2 = 0.22, Q2 = 0.01, CV-ANOVA p-value = 1.

Supplementary Figure 3. Comparison between the NSEIT and NSE group 3- to 15-month follow-up

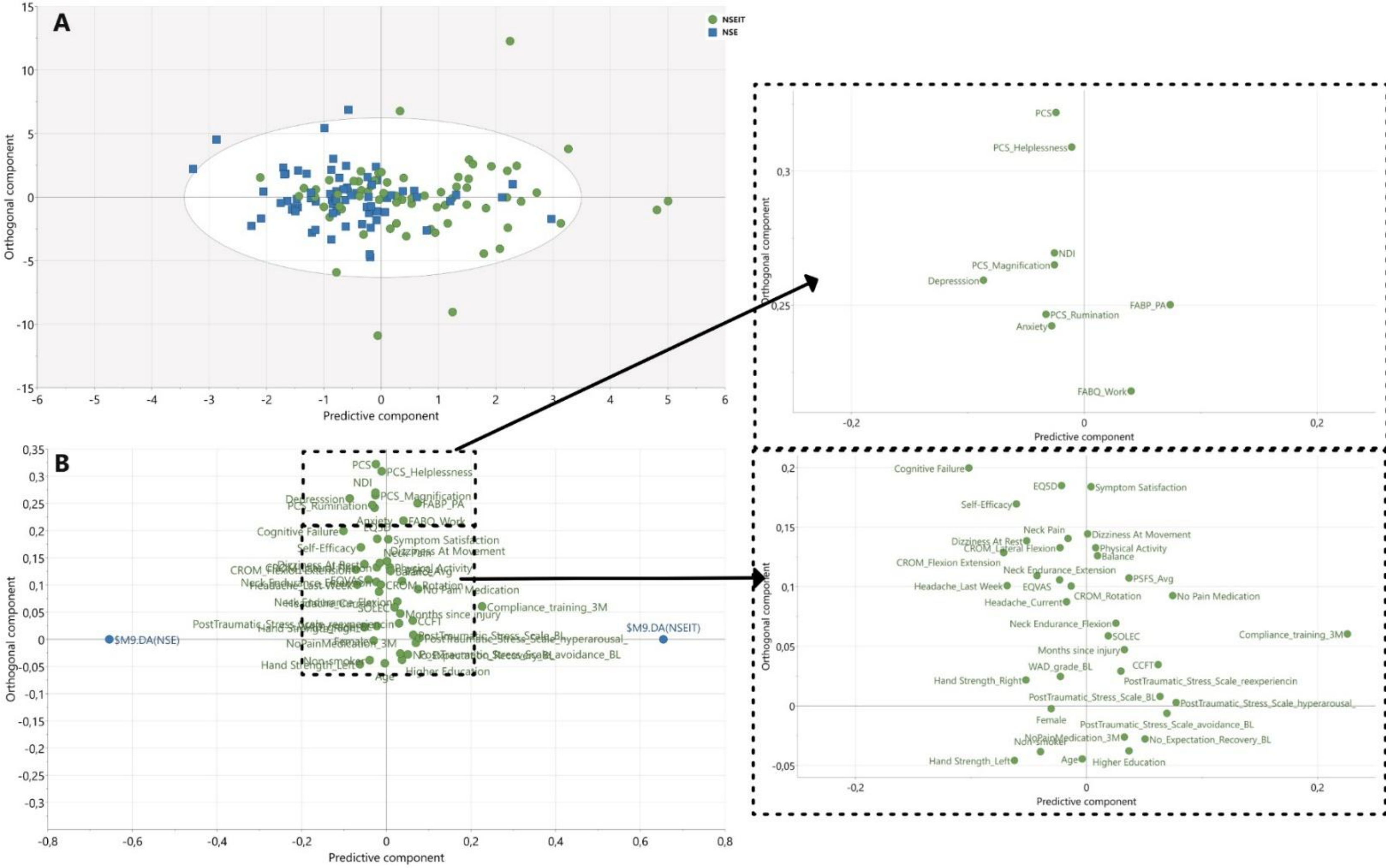

Orthogonal partial least squares discriminant analysis (OPLS-DA) model of NSE and NSEIT including the three dependent variables (NDI, neck pain and PSFSAVG) and the change score (baseline to 15 months) of all independent variables. No clear separation of the NSEIT and NSE groups could be seen. The x axis depicts the predictive component most important for separating groups, while the y axis represents the orthogonal component showing in-group variation, R2 = 0.17, Q2 = -0.10, CV-ANOVA p-value = 1.

Supplementary Figure 4. Comparison of patients with or without clinical improvement in Neck Disability Index (NDI) at 3 months follow-up based on baseline variables

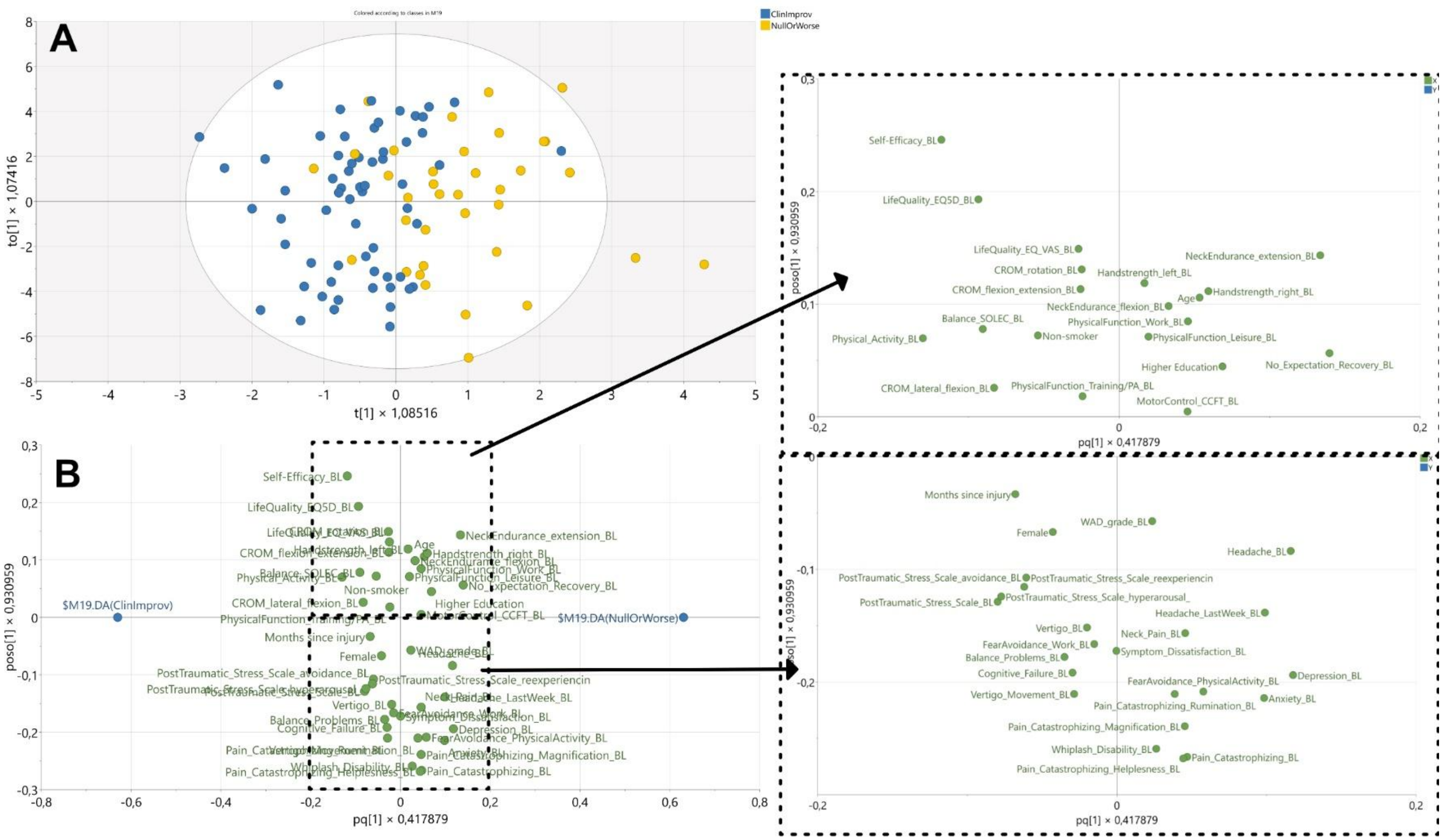

Orthogonal partial least squares discriminant analysis (OPLS-DA) model of clinically improved individuals (≥ 7% improvement in NDI) compared to individuals with non-effect/worse disability (≤ 0 % in NDI) at 3 months follow-up, based on the base-line variables. No clear separation of the improved vs not improved individuals could be seen, indicating no ability of predicting who will benefit regarding NDI with baseline values. The x axis depicts the predictive component most important for separating groups, while the y axis represents the orthogonal component showing in-group variation, $R2 = 0.38$, $Q2 = -0.17$, CV-ANOVA p-value = 1.

Supplementary Figure 5. Comparison of patients with or without clinical improvement in Neck Disability Index (NDI) at 15 months follow-up based on baseline variables

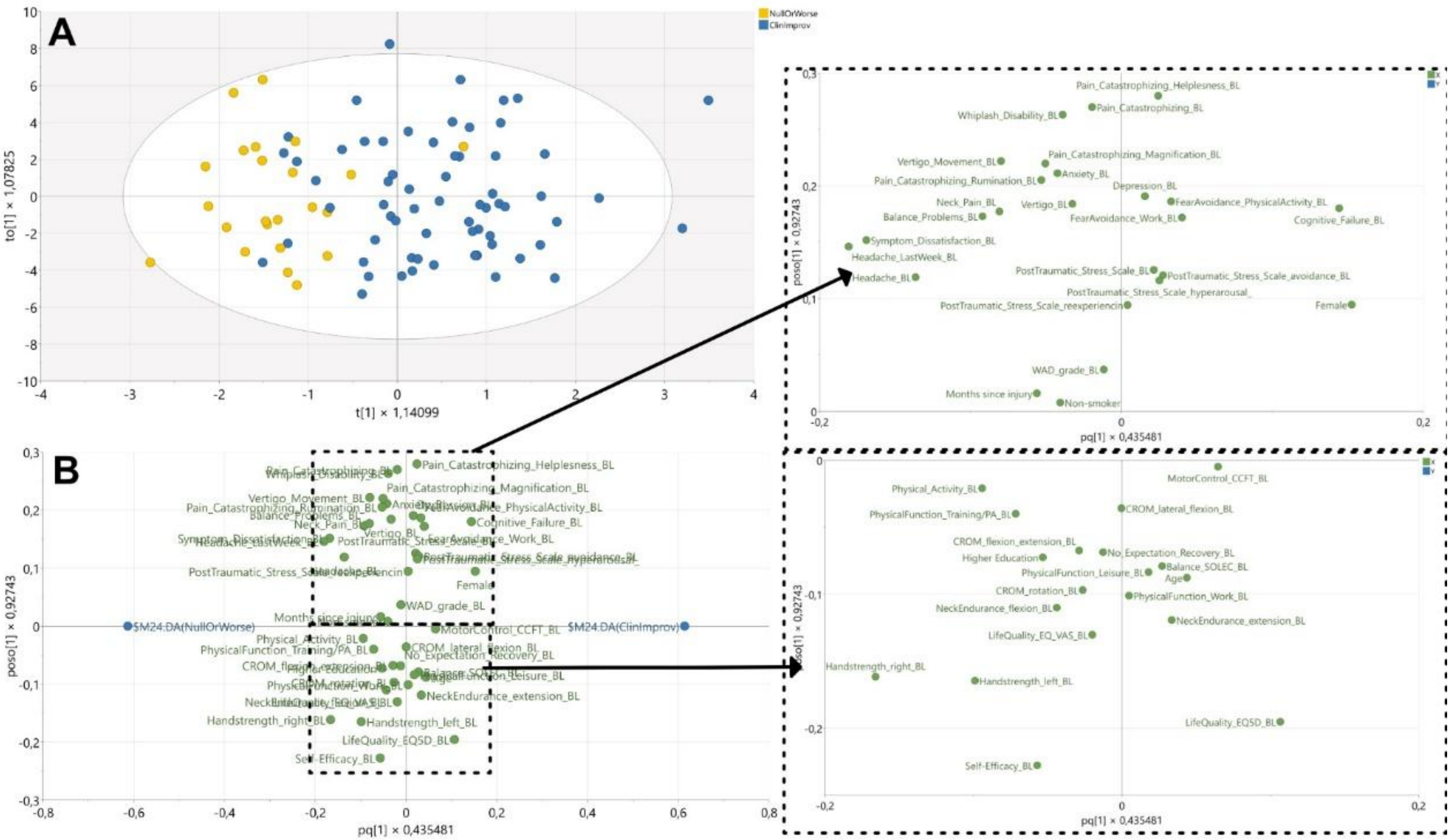

Orthogonal partial least squares discriminant analysis (OPLS-DA) model of clinically improved individuals (≥ 7% improvement in NDI) compared to individuals with non-effect/worse disability (≤ 0 % in NDI) at 15 months follow-up, based on the base-line variables. No clear separation of the improved vs not improved individuals could be seen, indicating no ability of predicting who will benefit regarding NDI with baseline values. The x axis depicts the predictive component most important for separating groups, while the y axis represents the orthogonal component showing in-group variation, R2 = 0.45, Q2 = -0.22, CV-ANOVA p-value = 1.

Supplementary Figure 6. Comparison of patients with or without clinical improvement in neck pain at 3 months follow-up based on baseline variables

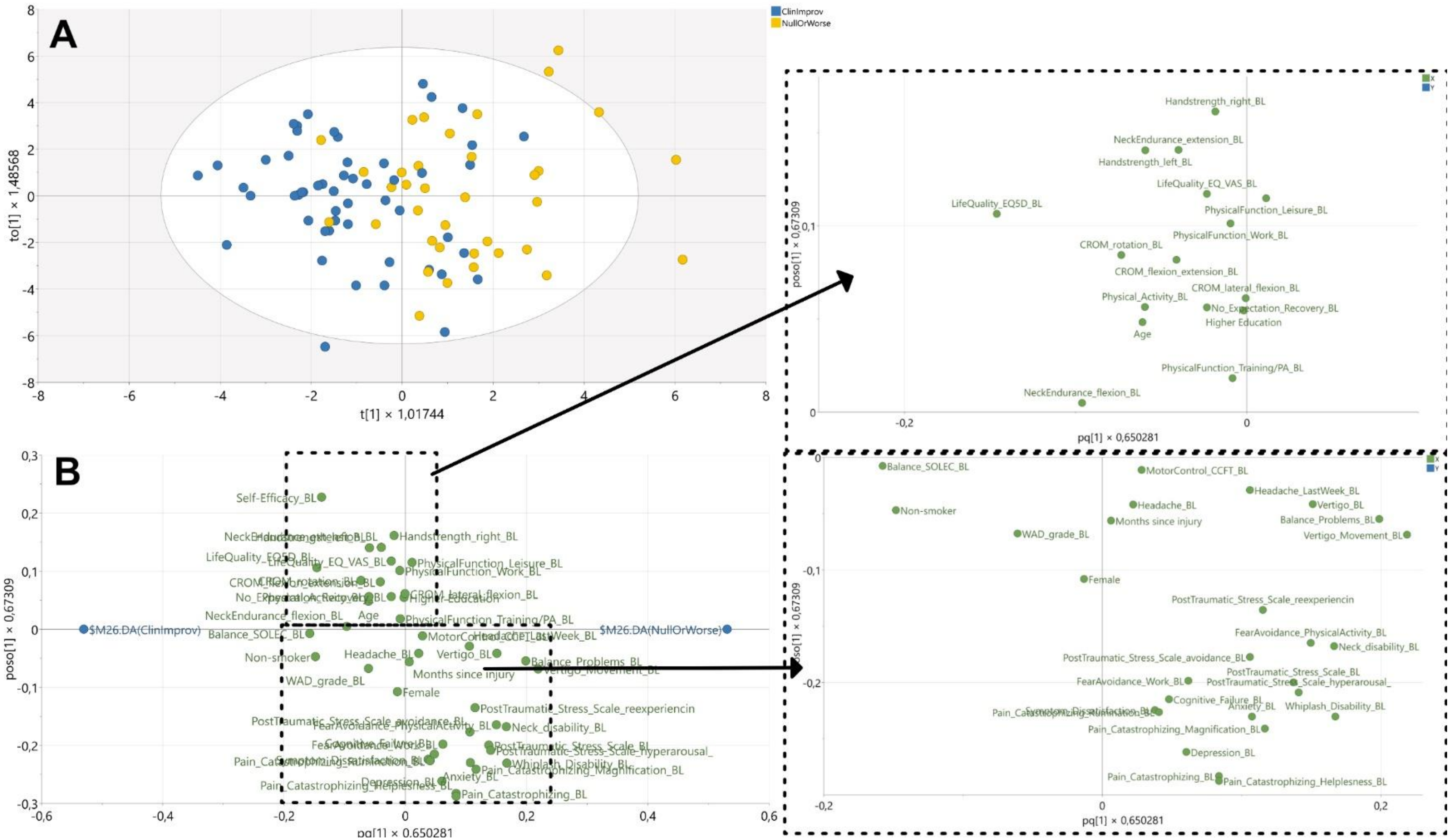

Orthogonal partial least squares discriminant analysis (OPLS-DA) model of clinically improved individuals (≥50% improvement in neck pain on Visual analogue Scale [VAS]) compared to individuals with non-effect/worsened pain (≤ 0 improvement in VAS) at 3 months follow-up, based on the base-line variables. No clear separation of the improved vs not improved individuals could be seen, indicating no ability of predicting who will benefit regarding neck pain with baseline values. The x axis depicts the predictive component most important for separating groups, while the y axis represents the orthogonal component showing in-group variation, R2 = 0.35, Q2 = -0.06, CV-ANOVA p-value = 1.

Supplementary Figure 7. Comparison of patients with or without clinical improvement in neck pain at 15 months follow-up based on baseline variables

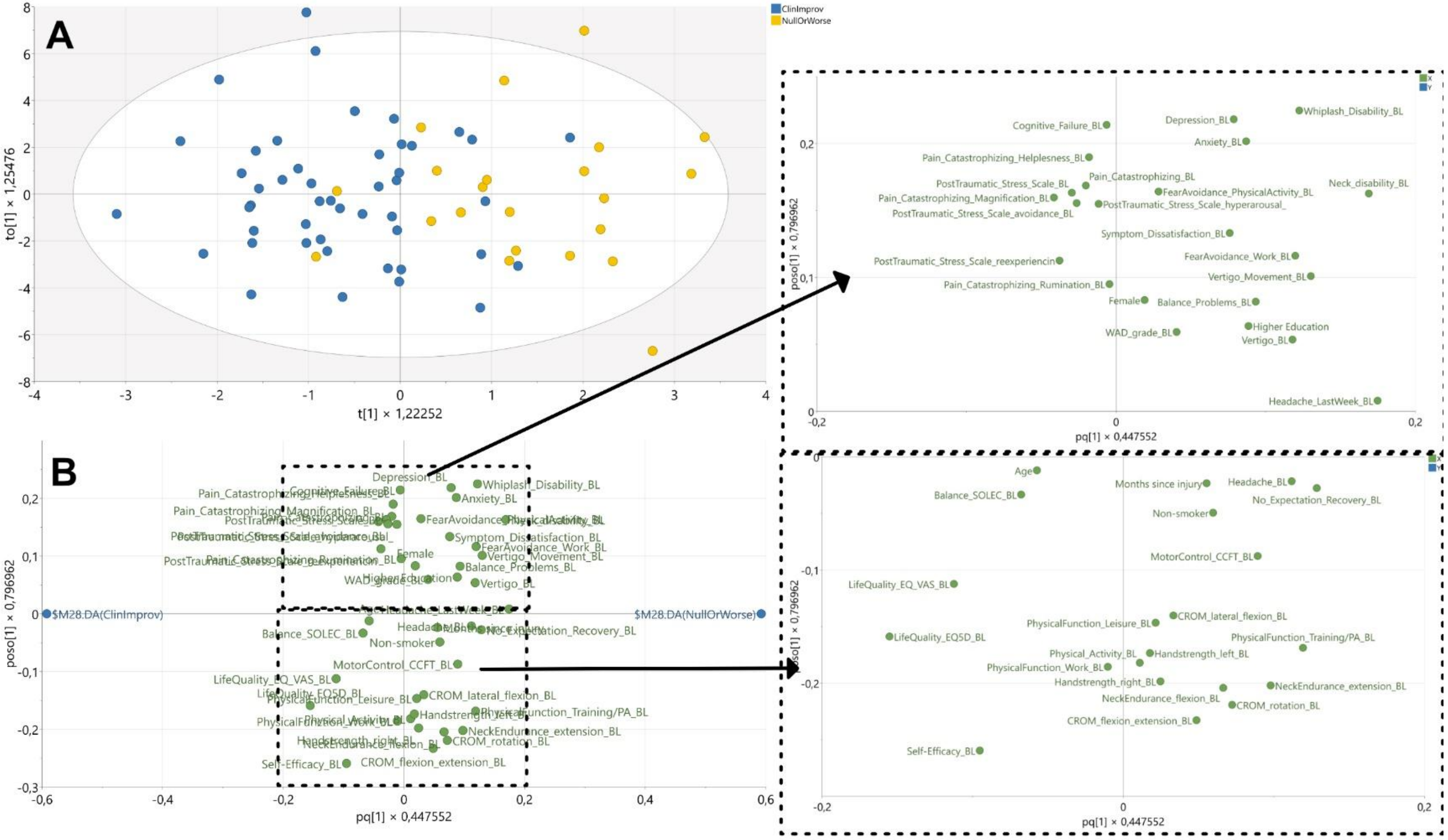

Orthogonal partial least squares discriminant analysis (OPLS-DA) model of clinically improved individuals (≥50% improvement in neck pain on Visual analogue Scale [VAS]) compared to individuals with non-effect/worsened pain (≤ 0 improvement in VAS) at 15 months follow-up, based on the base-line variables. No clear separation of the improved vs not improved individuals could be seen, indicating no ability of predicting who will benefit regarding neck pain with baseline values. The x axis depicts the predictive component most important for separating groups, while the y axis represents the orthogonal component showing in-group variation, R2 = 0.45, Q2 = -0.24, CV-ANOVA p-value = 1.

Supplementary Figure 8. Comparison of patients with or without clinical improvement in Patient Specific Functional Scale (PSFS) at 3 months follow-up based on baseline variables

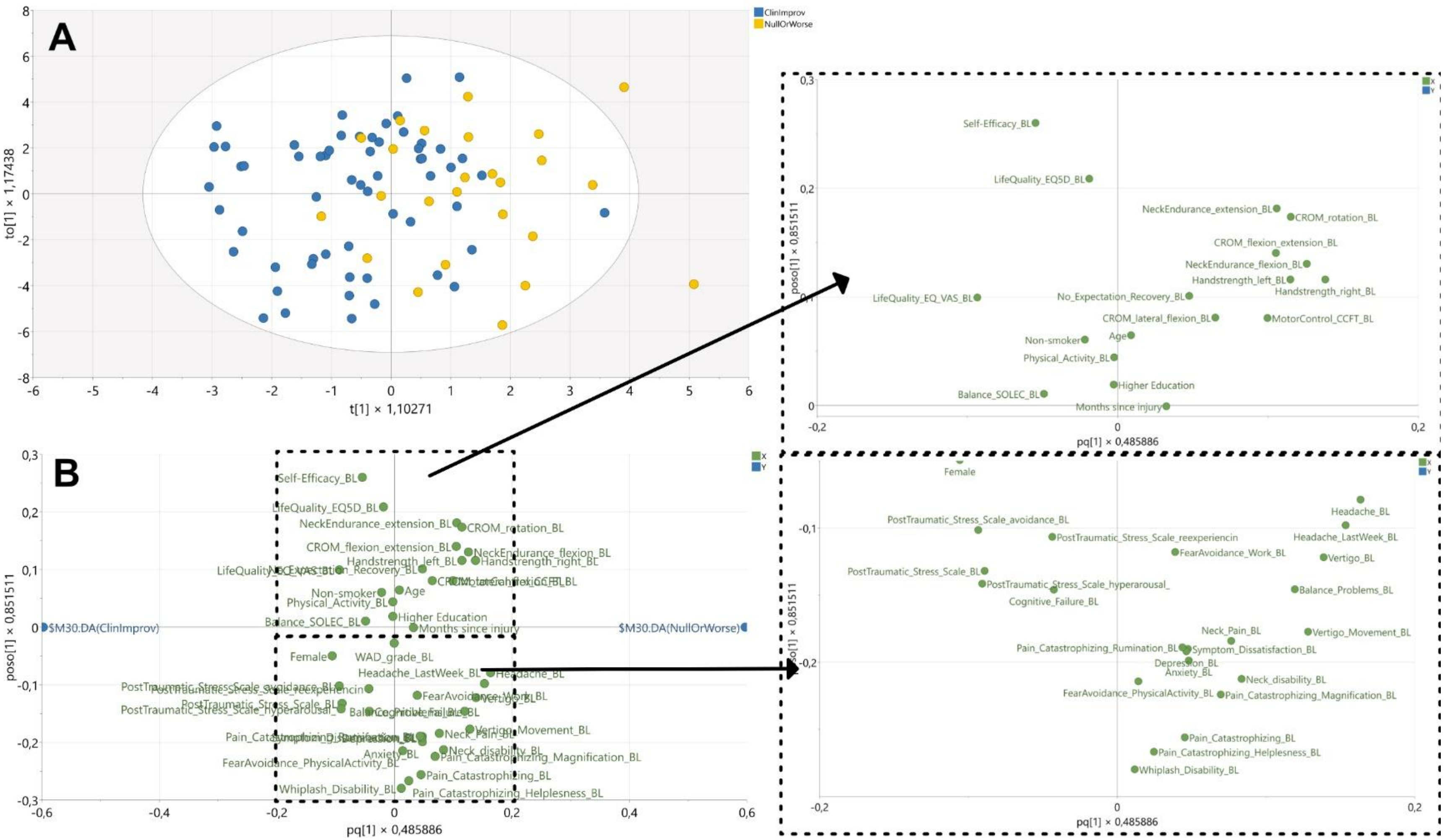

Orthogonal partial least squares discriminant analysis (OPLS-DA) model of clinically improved individuals (≥2 point improvement in PSFS) compared to individuals with non-effect/worsened pain (≤ 0 improvement in PSFS) at 3 months follow-up, based on the base-line variables. No clear separation of the improved vs not improved individuals could be seen, indicating no ability of predicting who will benefit regarding PSFS with baseline values. The x axis depicts the predictive component most important for separating groups, while the y axis represents the orthogonal component showing in-group variation, $R^2 = 0.30$, $Q^2 = -0.12$, CV-ANOVA p-value = 1.

Supplementary Figure 9. Comparison of patients with or without clinical improvement in Patient Specific Functional Scale (PSFS) at 15 months follow-up based on baseline variables

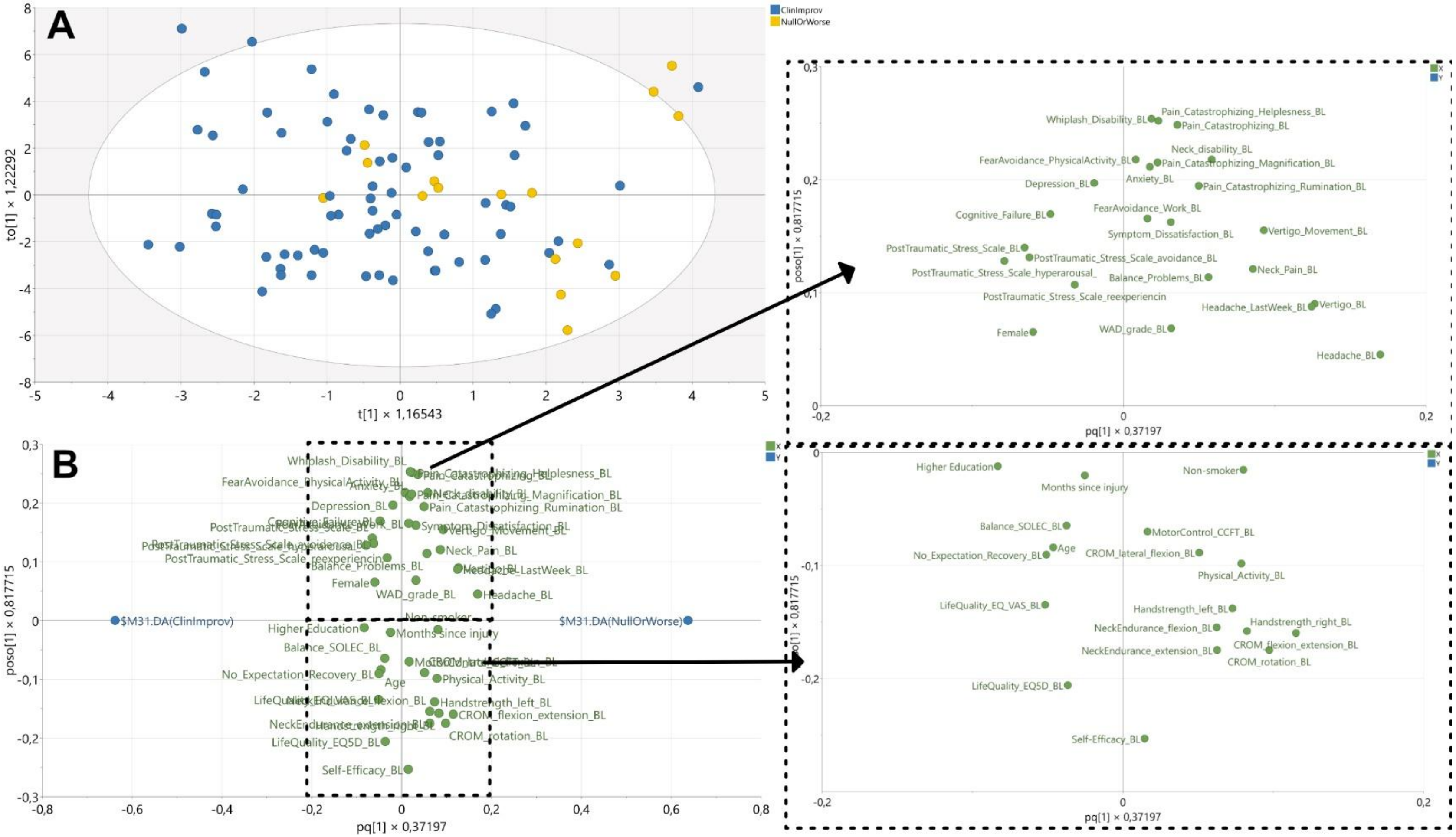

Orthogonal partial least squares discriminant analysis (OPLS-DA) model of clinically improved individuals (≥2 point improvement in PSFS) compared to individuals with non-effect/worsened pain (≤ 0 improvement in PSFS) at 15 months follow-up, based on the base-line variables. No clear separation of the improved vs not improved individuals could be seen, indicating no ability of predicting who will benefit regarding PSFS with baseline values. The x axis depicts the predictive component most important for separating groups, while the y axis represents the orthogonal component showing in-group variation, $R^2 = 0.19$, $Q^2 = -0.17$, CV-ANOVA p-value = 1.
